# Adherence to a healthy lifestyle in association with female infertility risk: the mediating role of uric acid

**DOI:** 10.3389/fnut.2025.1654495

**Published:** 2025-10-10

**Authors:** Zhihui Huang, Ziqi Yang, Lei King, Hong Chen, Xinxia Wan, Qimei Luo, Rili Hu, Lin Peng, Yan Zhao, Jialyu Huang

**Affiliations:** ^1^Jiangxi Key Laboratory of Reproductive Health, Jiangxi Maternal and Child Health Hospital, Jiangxi Branch of National Clinical Research Center for Obstetrics and Gynecology, Center for Reproductive Medicine, Nanchang Medical College, Nanchang, China; ^2^Department of Clinical Medicine, School of Queen Mary, Nanchang University, Nanchang, China; ^3^Bright Prosperity Institute, Hangzhou, China; ^4^Hubei Key Laboratory of Food Nutrition and Safety, MOE Key Lab of Environment and Health, Department of Nutrition and Food Hygiene, School of Public Health, Tongji Medical College, Huazhong University of Science and Technology, Wuhan, China; ^5^Department of Thyroid and Breast Surgery, Affiliated Hospital of North Sichuan Medical College, Nanchong, China

**Keywords:** lifestyle, infertility, uric acid, smoking, waist circumference

## Abstract

**Background:**

Infertility poses a significant health challenge for reproductive-aged women globally and could be shaped by both genetic risks and lifestyle factors. Existing studies predominantly focus on individual lifestyle components, while their joint effect on infertility remains limited.

**Methods:**

This large cross-sectional study analyzed data from 2,067 women aged 18–44 years, sourced from the National Health and Nutrition Examination Survey spanning 2013 to 2020. Six modifiable healthy lifestyle factors were included: current non-tobacco smoking, non-excessive alcohol drinking (1–14 g/day), sufficient physical activity (≥150 min/week), healthy diet (top 40% of Healthy Eating Index-2015 score), optimal waist circumference (<80 cm), and adequate sleep duration (7–9 h/day). Participants received 1 point for each factor (scale 0 to 6). Infertility status was self-reported in reproductive health questionnaire. Multivariable regression, stratified, sensitivity, and mediation analyses were performed. External validation was conducted on another 33,881 women enrolled from an academic reproductive medicine center in China from January 2014 to December 2022.

**Results:**

After adjusting for potential covariates, women with 5–6 healthy lifestyle factors exhibited a 70% lower risk of infertility (OR: 0.30, 95% CI: 0.17–0.52) compared to those with 0–1 factor. A 22% reduction in infertility risk was observed with each additional healthy lifestyle factor (OR: 0.78, 95% CI: 0.70–0.88). The association was more pronounced in those aged <30 years (*P*-interaction<0.001) and females who had never been pregnant (*P*-interaction = 0.01). Current nonsmoking and optimal waist circumference were identified as the most pivotal determinants among six factors. Additionally, serum uric acid was estimated to mediate 9.61% (95% CI: 3.93 to 21.88%) of the inverse relationship between the composite healthy lifestyle score and infertility. Serum uric acid levels were also confirmed to be positively associated with female infertility risk in real-world data analysis.

**Conclusion:**

Our study highlights the inverse association between adherence to a healthy lifestyle and female infertility via uric acid mediation. These findings provide supporting evidence that comprehensive lifestyle modification may be an effective, low-cost strategy for managing infertility.

## 1 Introduction

Infertility is clinically defined as the failure to conceive after at least 12 months of consistent, unprotected sexual activity. Recent estimates from the World Health Organization suggest that approximately 17.5% of people experience infertility in a lifetime (1), with the age-standardized prevalence rate of female infertility rising by 23.97% between 1990 and 2019 (2). Infertility not only presents challenges to conception but also contributes to significant psychological distress and social pressure (3). Furthermore, the increasing reliance on assisted reproductive technologies places a considerable financial burden on both patients and healthcare systems (4). Female infertility is also associated with an elevated risk of reproductive cancers, metabolic disorders, and cardiovascular diseases, highlighting its extensive implications for public health (5).

Given that fertility-related genetic predispositions are immutable (6), identifying modifiable factors that influence fecundability is critically important. In recent years, a healthy lifestyle has attracted growing attention as a cost-effective behavioral factor, due to its potential benefits in improving overall well-being (7). In epidemiological studies that examine individual health behaviors or exposures, other variables are often incorporated as covariates in statistical models. Under the context of female infertility, dietary patterns (8), body mass index (BMI) (9), tobacco smoking (10), alcohol intake (11), and physical activity (12) have garnered significant attention. Emerging lifestyle factors like sufficient sleep duration (13) and optimal waist circumference (14) are also accounted. The combination of various lifestyle factors has been linked to the onset of several conditions, including coronary heart disease (CHD), dementia, and diabetes (15–17). Nevertheless, only one prior study has examined a composite healthy lifestyle index in relation to female fecundability (18), and none have focused specifically on infertility.

Purine metabolism is significantly shaped by genetic risks and lifestyle factors (19). Uric acid, the end product of purine degradation, is considered a key biomarker for systemic oxidative stress and inflammation (20). Previous studies suggest that uric acid plays a role in several pathological processes linked to female reproductive disorders (21). Supporting this, an analysis including 2,884 women revealed an increased likelihood of infertility in individuals with elevated uric acid levels (22). Likewise, women with polycystic ovary syndrome (PCOS), hyperandrogenaemia, and endometriosis exhibit significantly higher serum uric acid concentrations (21, 23). These findings imply that uric acid may play a mediating role in the connection between healthy lifestyles and female infertility. However, the extent to which variability in uric acid contributes to this relationship remains underexplored.

To fill this gap, we constructed an extensive lifestyle scoring system to explore the relationship between infertility and lifestyle-related factors. Participants were assessed for adherence to a healthy lifestyle using an evaluation framework that includes six key modifiable factors: tobacco smoking, alcohol intake, physical activity, diet, waist circumference, and sleep duration. Furthermore, we investigated the role of uric acid as a mediator in this relationship, offering new perspectives on the underlying mechanisms and protective strategies.

## 2 Methods

### 2.1 Study population

The study utilized data from the National Health and Nutrition Examination Survey (NHANES), a biennial nationwide survey ethically approved and conducted by the National Center for Health Statistics to assess the health and nutritional status of the U.S. population (22). NHANES employs a multi-stage, stratified sampling method to ensure a representative sample, and collects data via surveys, clinical evaluations, and laboratory tests in accordance with the Declaration of Helsinki.

In the NHANES 2013–2020 cycles, 6,271 reproductive-aged females (18–44 years) were initially screened. Those who were pregnant or breastfeeding (*n* = 1,420), lacked infertility information (*n* = 549), or had missing lifestyle data (*n* = 1,944) were ruled out. Further exclusions were made for participants without serum uric acid measurements (*n* = 107) and those with absent covariates (*n* = 184). Finally, 2,067 participants were included in the analysis (Supplementary Figure S1).

### 2.2 Construction of healthy lifestyle score

The healthy lifestyle score was calculated by aggregating the total count of individual factors, including current non-tobacco smoking, non-excessive alcohol intake, sufficient physical activity, healthy diet, ideal waist circumference, and adequate sleep duration (24, 25). The healthy lifestyle score, whose higher values indicated healthier lifestyles, ranged from 0 to 6. According to previous reports, current non-tobacco smoking and consumption of 1–14 g/day alcohol were considered as healthy levels (24). For physical activity, moderate-to-vigorous leisure time physical activity of ≥150 min/week were defined as healthy level (24). Dietary information was gathered using a computerized 24-h dietary recall method, where the first recall was carried out through in-person interviews while the second was conducted via telephone. Healthy Eating Index-2015 (HEI-2015), which consists of 9 adequacy and 4 moderation components, was applied to evaluate diet quality. Healthy diet was characterized by being in the highest 40% of the HEI-2015 score (24). In this study, waist circumference served as an indicator of obesity, with a healthy measurement considered to be under 80 cm for women (26). Sufficient sleep duration was defined as sleeping 7–9 h per day (25). Detailed definitions and cut-off values for each component are provided in Supplementary Table S1.

### 2.3 Definitions of infertility

Infertility was ascertained with a self-reported reproductive health questionnaire (27). Specifically, participants were asked “Have you ever attempted to become pregnant over a period of at least a year without being pregnant?” and “Have you ever been to a doctor or other medical provider because you have been unable to become pregnant?” Infertility was classified in women who responded “Yes” to either question.

### 2.4 Assessment of covariates

Demographic, socioeconomic, and medical information were collected with a computer-assisted personal interview system by trained interviewers. Race/ethnicity was categorized as non-Hispanic White or Black, Mexican American, and others. Marital status was categorized as married and others (e.g., living with partner). Self-reported education attainment was grouped as under high school, high school, and above high school. To measure income status, the poverty-income ratio (PIR) was determined by dividing the family (or individual) income by the poverty line applicable to the survey year. Blood pressure (BP) was examined with mercury sphygmomanometers by technicians in mobile center. Hypertension was characterized by a systolic BP of ≥140 mmHg, diastolic BP of ≥90 mmHg, a physician diagnosis, or current use of prescribed antihypertensive medication. Diabetes were identified based on fasting plasma glucose ≥126 mg/dL, 2-h plasma glucose ≥200 mg/dL in oral glucose tolerance test, hemoglobin A1C ≥ 6.5%, a physician diagnosis, or the use of insulin or oral hypoglycemic medication. Cardiovascular disease (CVD) was characterized as physician-diagnosed CHD, angina/angina pectoris, heart attack, or stroke. Regularity of menstrual cycle, history of pelvic infection, history of pregnancy, and hormone pills usage were collected in reproductive health questionnaire.

### 2.5 Statistical methods

As the number of participants adhering to 0 or 6 factors was limited, those with 0–1 and 5–6 healthy lifestyle factors were combined to enhance statistical power (Table 1). After checking the distribution pattern with Q-Q plots, continuous data were presented as mean with standard deviation (SD), while categorical variables were presented as numbers with percentages. Population characteristics across healthy lifestyle scores were compared with one way analysis of variance and chi-square test for continuous and categorical data, respectively.

**Table 1:** Characteristics of study participants.

Characteristics^a^	Overall (*N*=2067)
Age, years	32.64 (7.33)
BMI, kg/m^2^	30.19 (8.79)
Waist circumference, cm	96.97 (19.51)
HEI-2015	49.69 (11.82)
Serum uric acid, mg/dL	4.55 (1.10)
Race/ethnicity, *n* (%)	
Non-Hispanic White	747 (36.14)
Non-Hispanic Black	493 (23.85)
Mexican American	299 (14.47)
Others	528 (25.54)
Marital status, *n* (%)	
Married	943 (45.62)
Others	1124 (54.38)
Education attainment, *n* (%)	
Under high school	252 (12.19)
High school	387 (18.72)
Above high school	1428 (69.09)
Family PIR, *n* (%)	
<1.3	697 (33.72)
1.3-<3.5	779 (37.69)
≥3.5	591 (28.59)
Current nonsmoking, *n* (%)	1651 (79.87)
Low-to-moderate alcohol drinking, *n* (%)	1740 (84.18)
Adequate physical activity, *n* (%)	850 (41.12)
Healthy diet, *n* (%)	827 (40.01)
Optimal waist circumference, *n* (%)	426 (20.61)
Sufficient sleep duration, *n* (%)	1345 (65.07)
No. of healthy lifestyle factors, *n* (%)	
0	18 (0.87)
1	158 (7.64)
2	374 (18.09)
3	600 (29.03)
4	529 (25.59)
5	311 (15.05)
6	77 (3.73)
Hypertension, *n* (%)	366 (17.71)
Diabetes, *n* (%)	151 (7.31)
CVD, *n* (%)	45 (2.18)
Regular menstrual cycle, *n* (%)	1862 (90.08)
History of pelvic infection, *n* (%)	107 (5.18)
History of pregnancy, *n* (%)	1432 (69.28)
Taking hormone pills, *n* (%)	87 (4.21)

a Continuous variables were expressed as mean (SD), and categorical variables were presented as number (percentage).

BMI, body mass index; CVD, cardiovascular disease; HEI, Healthy Eating Index; PIR, poverty-income ratio.

Multivariable logistic regression analysis was first conducted to examine the independent healthy lifestyle-infertility association. Multicollinearity was excluded since all variance inflation factors were below 10. In model 1, we accounted for age (categorized as below 30 or above) and race/ethnicity (classified as non-Hispanic White and other groups). In model 2, we additionally controlled for marital status (categorized as married or others), PIR (categorized as below 3.5 or above), education level (classified as above high school and high school or below), as well as hypertension, diabetes, CVD, regular menstrual cycle, history of pelvic infection, history of pregnancy, and use of hormone pills (all recorded as yes or no). For each additional healthy lifestyle factor, we also estimated its association with infertility by computing the multivariable-adjusted odds ratios (ORs) and 95% confidence intervals (CIs). To examine whether the aforementioned confounders modified the relationship, we further conducted stratified analyses and evaluated multiplicative interactions with likelihood ratio tests. To investigate the individual contribution of six lifestyle factors, we initially evaluated one factor at a time with all other factors adjusted simultaneously. Then, we reconstructed new scores by removing one healthy lifestyle factor each time from the score and adjusted the removed factor in the models.

The following sensitivity analyses were carried out to assess the robustness of results. First, women who reported having underwent an ovariectomy or hysterectomy were excluded. Second, we redefined the healthy level of alcohol intake as none to moderate alcohol drinking (≤14 g/day). Third, propensity score (PS) adjustment, another method to control for covariates (28), was utilized to address observed confounding. Fourth, the potential for residual confounding was assessed by calculating *E*-values, which estimate the minimum association strength that an unaccounted confounder must have with both exposure and outcome to explain away the observed relationship after adjusting for measured variables (29). Finally, we developed a weighted healthy lifestyle score system to modify the combined pattern. Briefly, the *β* coefficients were computed from a logistic regression model that accounted for all six lifestyle factors and relevant covariates. Each binary lifestyle factor (0 or 1) was then multiplied by its corresponding β coefficients with weighted standardization, summed, and finally multiplied by 6. We then categorized the weighted lifestyle scores into quartiles to evaluate their association with infertility risk. A restricted cubic spline (RCS) with three knots was also constructed to illustrate the dose–response relationship.

The association of healthy lifestyle score with serum uric acid was examined with a generalized linear regression model. A multivariable-adjusted logistic regression model and RCS were used to investigate the association of serum uric acid with risk of infertility. Mediation analysis was implemented to assess the mediating role of uric acid in the association between the healthy lifestyle score and infertility, utilizing the R “mediation” package (version 4.5.0).

Survey weights provided by NHANES were not applied in our analyses. This decision was made because some of the statistical models used (e.g., mediation analysis) are not compatible with complex survey weights, and for consistency we therefore reported unweighted results throughout. All statistical analyses were conducted with R version 4.3.2 (The R Foundation for Statistical Computing, Vienna, Austria). Two-sided *p*-values < 0.05 were considered statistically significant.

## 3 Results

### 3.1 Population characteristics

This cross-sectional study comprised 2,067 reproductive-aged women (mean age 32.64 years) (Table 1). The proportion of individuals engaging in key healthy lifestyle behaviors were as follow: current non-tabacco smoking (79.87%), low-to-moderate alcohol intake (84.18%), sufficient physical activity (41.12%), healthy diet (40.01%), optimal waist circumference (20.61%), and adequate sleep duration (65.07%) (Table 1). The distribution of population characteristics is shown in Supplementary Table S2. Females with more healthy lifestyle factors were more likely to be younger, other races/ethnics, married, well-educated, and had better income status, higher HEI-2015 scores and lower BMI, waist circumference, and serum uric acid level. In contrast, women with fewer healthy lifestyle factors were more inclined to be hypertensive and diabetic. The prevalence of CVD, irregular menstrual cycle, and pelvic infection were also higher in individuals with a reduced number of healthy lifestyle factors (Supplementary Table S2).

### 3.2 Association of combined healthy lifestyle score with risk of infertility

In the crude model, the OR for infertility among participants with 5–6 *versus* 0–1 healthy lifestyle factors was 0.34 (95% CI: 0.20–0.55) (Table 2). After fully adjusting for confounding factors, women with 5–6 healthy lifestyle factors were confronted with 70% (OR: 0.30, 91% CI: 0.17–0.52) lower risk of infertility when compared to those with 0–1 factor (Table 2). In addition, each additional healthy lifestyle factor was associated with 22% (OR: 0.78, 95% CI: 0.70–0.88) decreased risk of infertility (Table 2).

**Table 2:** Association of healthy lifestyle score with risk of infertility.

Variable	No. of healthy lifestyle factors					Each additional healthy lifestyle factor
	0–1	2	3	4	5–6	
Case/total (%)	40/176 (22.73)	61/374 (16.31)	83/600 (13.83)	73/529 (13.8)	35/388 (9.02)	
Crude model	1.00 (reference)	0.66 (0.43-1.04)	0.55 (0.36-0.84)	0.54 (0.36-0.84)	0.34 (0.20-0.55)	0.80 (0.73-0.89)
*P*-value		0.071	0.005	0.006	<0.001	<0.001
Model 1^a^	1.00 (reference)	0.68 (0.44-1.07)	0.56 (0.37-0.87)	0.57 (0.37-0.89)	0.37 (0.22-0.61)	0.82 (0.74-0.90)
*P*-value		0.095	0.008	0.011	<0.001	<0.001
Model 2^b^	1.00 (reference)	0.60 (0.38-0.96)	0.49 (0.31-0.76)	0.49 (0.30-0.78)	0.30 (0.17-0.52)	0.78 (0.70-0.88)
*P*-value		0.033	0.002	0.003	<0.001	<0.001

a Model 1 was adjusted for age (<30, ≥30 years) and race/ethnicity (non-Hispanic White, others).

b Model 2 was further adjusted for marital status (married, others), family poverty-income ratio (<3.5, ≥3.5), education attainment (above high school, high school and below), hypertension (yes, no), diabetes (yes, no), CVD (yes, no), regular menstrual cycle (yes, no), history of pelvic infection (yes, no), history of pregnancy (yes, no), and taking hormone pills (yes, no).

### 3.3 Stratified, interaction, and sensitivity analyses

To assess whether the healthy lifestyle-infertility relationship differed by demographic, socioeconomic, and medical status, we conducted stratified analyses and examined interaction effects (Table 3). The inverse association of healthy lifestyle score with infertility was found to be stronger in those aged <30 years (*P*-interaction <0.001) and females who have never been pregnant (*P*-interaction = 0.01). No significant interactions were observed regarding other subgroups.

**Table 3:** Association of healthy lifestyle score with infertility stratified by confounders.

Confounders	No. of subjects	OR (95% CI)^a^	*P*-value	*P*-interaction
Overall	2067	0.78 (0.70-0.88)	<0.001	
Age, years				<0.001
<30	767	0.60 (0.47-0.75)	<0.001	
≥30	1300	0.85 (0.75-0.97)	0.019	
Race/ethnicity				0.06
Others	1320	0.71 (0.61-0.82)	<0.001	
Non-Hispanic White	747	0.91 (0.76-1.08)	0.269	
Marital status				0.11
Others	1124	0.76 (0.63-0.90)	0.002	
Married	943	0.81 (0.70-0.94)	0.005	
Family PIR				0.30
<3.5	1476	0.75 (0.66-0.86)	<0.001	
≥3.5	591	0.85 (0.69-1.05)	0.137	
Education attainment				0.86
High school and below	639	0.77 (0.64-0.93)	0.009	
Above high school	1428	0.78 (0.68-0.90)	0.001	
Hypertension				0.31
No	1701	0.77 (0.68-0.88)	<0.001	
Yes	366	0.84 (0.65-1.06)	0.150	
Diabetes				0.91
No	1996	0.80 (0.69-0.92)	<0.001	
Yes	158	0.63 (0.39-1.02)	0.207	
CVD				0.85
No	1916	0.79 (0.70-0.88)	<0.001	
Yes	151	0.78 (0.52-1.15)	0.088	
Regular menstrual cycle				0.18
No	205	0.56 (0.38-0.80)	0.002	
Yes	1862	0.82 (0.73-0.92)	0.001	
History of pelvic infection				0.97
No	1960	0.78 (0.70-0.88)	<0.001	
Yes	107	0.80 (0.49-1.26)	0.337	
History of pregnancy				0.01
No	635	0.72 (0.56-0.93)	0.012	
Yes	1432	0.82 (0.72-0.93)	0.002	
Taking hormone pills				0.86
No	1980	0.78 (0.69-0.88)	<0.001	
Yes	87	0.90 (0.56-1.42)	0.650	

a Data were presented as odds ratio (95% confidence interval) associated with each additional healthy lifestyle factor. Models were adjusted for age (<30, ≥30 years), race/ethnicity (non-Hispanic White, others), marital status (married, others), family poverty-income ratio (<3.5, ≥3.5), education attainment (above high school, high school and below), hypertension (yes, no), diabetes (yes, no), CVD (yes, no), regular menstrual cycle (yes, no), history of pelvic infection (yes, no), history of pregnancy (yes, no), and taking hormone pills (yes, no).

CI, confidence interval; OR, odds ratio.

Among individual lifestyle factors, only being a current non-smoker and having an optimal waist circumference were significantly associated with a reduced risk of infertility. The ORs (95% CIs) were 0.70 (0.51–0.98) and 0.61 (0.41–0.89), respectively. (Supplementary Table S3). Marginally significant and inverse association between low-to-moderate drinking and infertility was also observed (OR: 0.72, 95% CI: 0.53–1.00) (Supplementary Table S3). The associations of five-component lifestyle scores with infertility were attenuated when current non-tobacco smoking, non-excessive drinking, sufficient physical activity, health-conscious diet, ideal waist circumference, and adequate sleep duration were removed from the score, with ORs (95% CIs) comparing 4–5 versus 0–1 healthy lifestyle factors being 0.47 (0.29–0.74), 0.49 (0.31–0.76), 0.46 (0.29–0.72), 0.36 (0.23–0.57), 0.43 (0.28–0.68), and 0.37 (0.23–0.57), respectively (Supplementary Table S4).

**Figure 1:**
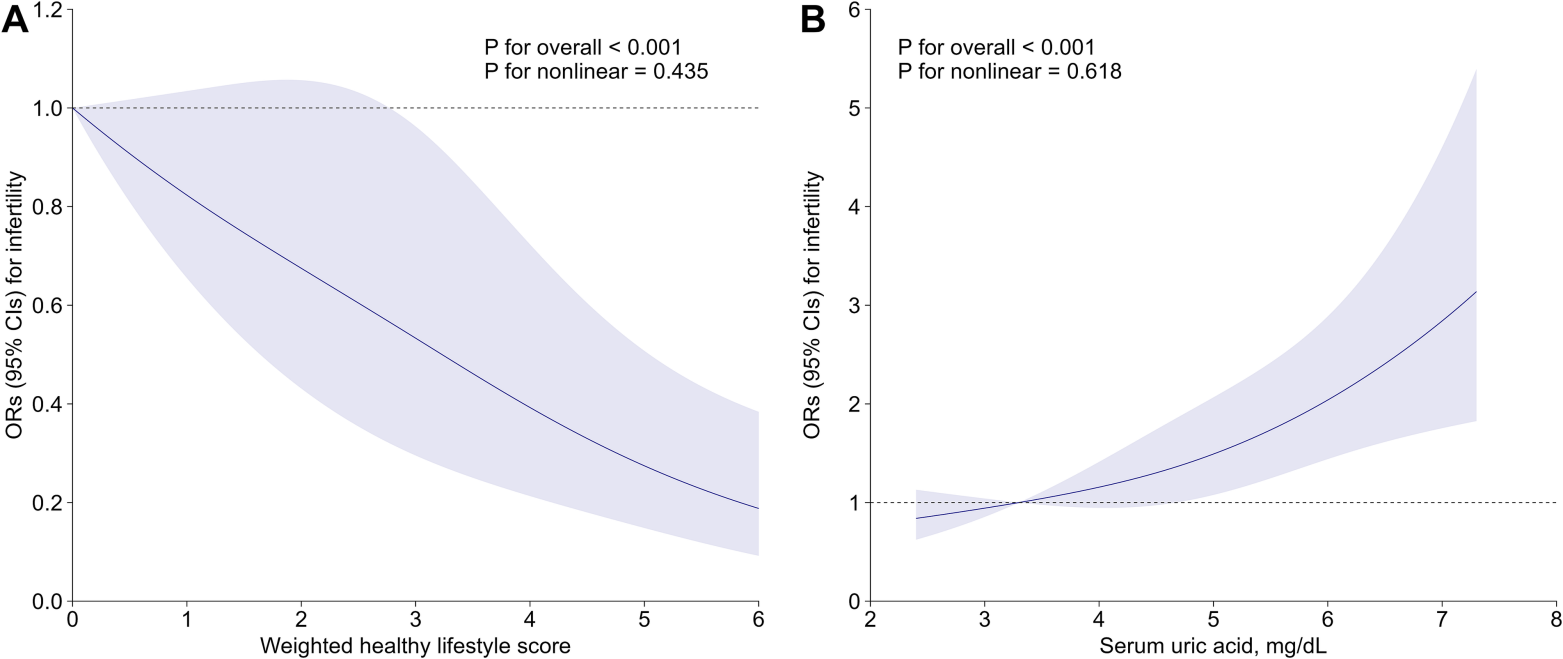
Associations of **(A)** weighted healthy lifestyle score and **(B)** serum uric acid with risk of infertility. Lines represent multivariable-adjusted OR, and shaded areas represent 95% CI. Models were adjusted for age, race/ethnicity, marital status, poverty-income ratio, education level, hypertension, diabetes, cardiovascular disease, regular menstrual cycle, history of pelvic infection, history of pregnancy, and taking hormone pills. CI, confidence interval; OR, odds ratio.

Multiple sensitivity analyses were carried out to test the consistency of our findings (Supplementary Tables S5–S10). After excluding women with an ovariectomy or hysterectomy history, those with 5–6 healthy lifestyle factors exhibited a 69% lower risk of infertility (OR: 0.31, 91% CI: 0.18–0.55) compared to those with only 0–1 factor (Supplementary Table S5). The inverse association persisted in sensitivity analysis redefining the healthy level of alcohol drinking, with an OR of 0.78 (95% CI: 0.69–0.87) for infertility per additional healthy lifestyle factor (Supplementary Table S6). After applying PS adjustment to cope with observed confounders, the OR for infertility comparing participants with 5–6 *versus* 0–1 healthy lifestyle factors was 0.35 (95% CI: 0.21–0.58) (Supplementary Table S7). The *E*-value was as high as 6.12 (Supplementary Table S8), indicating that only exceptionally strong confounding factors negate the observed inverse association. Finally, in the weighted healthy lifestyle score model, optimal waist circumference was found to have the largest contribution (weighted *β*: 0.30), followed by current non-tobacco smoking (weighted β: 0.22), non-excessive alcohol intake (weighted β: 0.20), sufficient physical activity (weighted β: 0.14), adequate sleep duration (weighted β: 0.08), and healthy diet (weighted β: 0.06) (Supplementary Table S9). After controlling for all covariates, females with the highest weighted healthy lifestyle score quartile were confronted with 56% (OR: 0.44, 95% CI: 0.29–0.66) decreased risk of infertility compared to those in the lowest quartile (Supplementary Table S10). The RCS further demonstrated a negative dose–response relationship between the weighted score and infertility prevalence, with a significant overall trend (*p* < 0.001) and no evidence of nonlinearity (*p* = 0.435) (Figure 1A).

### 3.4 Mediation analyses

**Figure 2:**
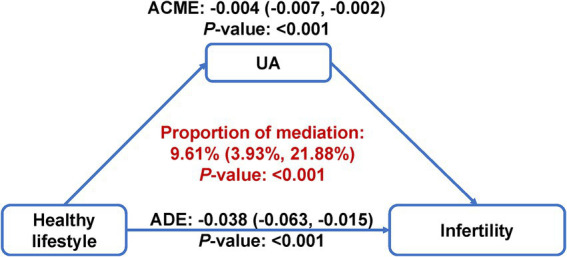
Mediation analysis of serum uric acid on the association of healthy lifestyle score with risk of infertility. The mediation model was adjusted for age, race/ethnicity, marital status, poverty-income ratio, education level, hypertension, diabetes, cardiovascular disease, regular menstrual cycle, history of pelvic infection, history of pregnancy, and taking hormone pills. ACME, average causal mediation effect; ADE, average direct effect; UA, uric acid.

After adjusting for all covariates, subjects with 5–6 healthy lifestyle factors had significantly lower serum uric acid measurements as compared to those with 0–1 healthy lifestyle factors (β: –0.35, 95% CI: −0.55 to −0.15) (Supplementary Table S11). The multivariable-adjusted OR (95% CI) for infertility comparing extreme uric acid quartiles was 1.98 (1.39–2.85) (Supplementary Table S12). Per SD increment in serum uric acid was associated with a 31% (OR: 1.31; 95% CI: 1.16–1.49) elevated risk of infertility (Supplementary Table S12). RCS analysis consistently indicated a strong positive and linear association between serum uric acid and infertility (*P*-overall<0.001, *P*-nonlinearity = 0.618) (Figure 1B). As further depicted in Figure 2, uric acid was found to mediate 9.61% (95% CI: 3.93 to 21.88%) of the inverse healthy lifestyle score-infertility association.

### 3.5 External validation analyses

To further validate the association between serum uric acid concentration and female infertility, a real-world data analysis was performed at the Center for Reproductive Medicine, Jiangxi Maternal and Child Health Hospital with ethical approval (No. 2024–11). A total of 41,289 women were enrolled from January 2014 to December 2022, of whom 92 were above 45 years old, 707 lacked infertility duration data, 2,645 had infertility due to male factor only, 3,701 had no serum uric acid measurements, and 263 had absent covariates. Finally, 33,881 participants were included in the analysis, and 32,027 (94.5%) were diagnosed with infertility, confirmed as ≥12 months of unsuccessful attempts to conceive without contraception (Supplementary Table S13).

Consistently, women with higher serum uric acid levels were found to have higher odds of infertility (*p* = 0.002) (Supplementary Table S14). After adjusting for covariates such as age, gravidity, parity, smoking status, systolic and diastolic BP, and year of treatment, the positive association remained statistically significant (*p* = 0.007). When categorized into quartiles, the risk of infertility was ~1.2 times greater among women with serum uric acid levels >328 μmol/L (Q4) than those ≤ 248 μmol/L (Q1) in both crude (OR: 1.21, 95% CI: 1.06–1.39) and adjusted (OR: 1.19, 95% CI: 1.04–1.37) models (Supplementary Table S14).

## 4 Discussion

The present study provides the first integrated evaluation of the relationship between comprehensive lifestyle factors and infertility, incorporating both traditional and emerging modifiable behaviors. Women who adopted more healthy lifestyle factors showed a significantly lower infertility risk, with optimal waist circumference and current nonsmoking identified as the most pivotal determinants among six factors. Moreover, uric acid mediated 9.61% of the inverse association between healthy lifestyle and infertility. These findings provide supporting evidence that the adoption of holistic and targeted lifestyle strategies may be an effective, low-cost approach associated with lower infertility risk and improved public health outcomes.

Our study reported that women with 5–6 healthy lifestyle factors had a 70% lower infertility risk than those with 0–1 factor, with each additional factor associated with decreased risk by 22%. Sensitivity analysis using a weighted score also yielded results nearly identical to those of the unweighted model. To the best of our knowledge, prior researches have not explored the specific relationship between this composite metric of the six lifestyle factors and infertility prevalence, as they predominantly concentrated on individual lifestyle components. Nonetheless, a prospective study conducted in Singapore similarly proposed a risk-scoring system based on six modifiable factors—BMI, dietary habits, smoking status, alcohol consumption, folic acid supplementation, and maternal age—to evaluate their association with fecundability. Each additional risk factor was associated with a 23% decrease in the probability of conception within a menstrual cycle (18). The study was strengthened by the cohort design, but was limited in the sample size of 937 reproductive-aged Asian women only. With a larger and ethnically diverse population, our study bridges this gap and further confirms the role of healthy lifestyle in fertility through a more comprehensive scoring framework (24, 25).

The stratified analysis revealed a stronger inverse association between healthy lifestyle scores and infertility risk in younger and nulligravid women. For females, the quantity and quality of oocytes begin to decline significantly starting in their mid-30s, and this decline accelerates as they approach their late 30s and early 40s (30). Thus, the impact of aging on ovarian reserve depletion may be hard to offset by the influence of modifiable lifestyle factors. Infertility can be categorized into primary infertility, pertaining to nulligravid women who have not attained a pregnancy, and secondary infertility, characterized by the inability to conceive subsequent to a previous pregnancy (31). Therefore, secondary infertility may arise from complications related to prior pregnancies, such as intrauterine adhesion. In this regard, healthy lifestyle practices may benefit primary infertility but exhibit relatively restricted efficacy in addressing secondary infertility with pathological structural alterations. Overall, these findings suggest that lifestyle modification may be especially effective when adopted early in the reproductive lifespan and in women at risk of primary infertility, further supporting the integration of lifestyle assessment into both fertility care and preconception counseling for these subgroups.

Among the six factors we analyzed, optimal waist circumference and smoking were identified as the most significant contributors to infertility risk. Waist circumference is a simple and practical anthropometric measure linked to abdominal fat distribution. Larger waist circumferences have been implicated in ovarian follicular membrane irregularities (32), elevated insulin and androgen levels (33), as well as chronic inflammation (34), all of which could lead to reproductive challenges. Clinical studies also showed that waist circumference acts as a key predictor of reduced fertility independent of BMI (14, 35), and associates with differences in ART outcomes (36, 37). In terms of smoking, it is recognized to exert a detrimental effect on female fertility (38). Tobacco smoke contains harmful compounds, including heavy metals like cadmium and lead, as well as reactive oxygen species such as superoxide and hydroxyl radicals (39). Nicotine lowers progesterone and estrogen levels, reduces blood flow, disrupts uterine and fallopian tube contractility, and even hinders cell proliferation and induces DNA damage (40). As a result, a large meta-analysis comparing 10,928 female smokers revealed that smokers exhibited a significantly elevated risk of infertility (OR: 1.60, 95% CI: 1.34–1.91) (41).

Additionally, we identified a marginally significant inverse association between non-excessive alcohol consumption (1–14 g/day) and infertility. When alcohol consumption was omitted from the lifestyle scoring system, the odds of infertility increased by 19%. Aligning with our research, a retrospective study involving 39,612 women found that those with moderate alcohol consumption experienced a shorter time to pregnancy compared to non-drinkers (42). Moreover, a study focusing on women undergoing artificial donor insemination showed slightly higher fecundability among those consuming 1–10 glasses of alcohol per week before insemination compared to abstainers (43). However, some studies have revealed that low-to-moderate levels of alcohol consumption are associated with increased infertility, while others observed no significant relationship (44). The inconsistent findings deserve further investigation with more confirmatory conclusion.

Women with higher healthy lifestyle scores exhibited significantly lower serum uric acid levels (*β*: –0.35, 95% CI: −0.55 to −0.15). Conversely, each standard deviation increase in serum uric acid was associated with a 31% higher risk of infertility. Substantial evidence, including the real-world data analysis in the present study, have indicated that elevated serum uric acid levels are associated with a higher likelihood of female infertility and infertility-related diseases, such as PCOS and endometriosis (21, 22). Proposed mechanisms include oxidative stress, mitochondrial dysfunction and chronic inflammation which disrupt hormonal homeostasis and metabolic regulation (21). Uric acid may trigger an inflammatory response that produces interleukin-1β, which interferes with endometrial receptivity and impedes embryo implantation (21, 45). Additionally, high uric acid levels are correlated with a greater likelihood of anovulation and disruptions in oocyte maturation (46). Therefore, we propose that serum uric acid levels may partially mediate the inverse association between a healthy lifestyle and female infertility. However, the modest proportion mediated (9.61%) suggests that additional mediators, such as insulin resistance, lipid metabolism, and hormonal milieu, may also contribute to the lifestyle-infertility relationship (47). Accordingly, future work should evaluate multiple-mediator models and longitudinal data to delineate pathway contributions. In addition, the mediation analysis relies on several key assumptions, including no unmeasured mediator-outcome confounding, no unmeasured exposure-mediator confounding, and no exposure-induced mediator-outcome confounding. While these assumptions are standard, they may not be fully testable in an observational study, and violations could bias the proportion mediated. Therefore, our results should be interpreted as supportive evidence rather than definitive proof of causal pathways.

The major strength of our study is the systematic evaluation of multiple healthy lifestyle factors and female infertility using a population-based dataset. Several statistical models, including multivariable regression, stratified, and sensitivity analyses, were applied to validate the stability and reliability of results. However, several limitations should be admitted. First, the cross-sectional nature of the data prevents causal inferences and reverse causation cannot be excluded. Infertility itself may influence behaviors such as smoking cessation, dietary adjustments, or weight management, which complicates the interpretation of lifestyle–infertility associations. Prospective longitudinal studies are thus needed to confirm the temporal and causal relationships. Second, the reliance on self-reported reproductive and lifestyle questionnaires may introduce misclassification or reporting bias, which could potentially affect true associations. However, NHANES instruments have undergone validation against objective reference methods (48, 49), supporting the general reliability of these measures. Third, the 2019–2020 NHANES cycle overlapped with the COVID-19 pandemic, during which significant changes in diet, physical activity, and other lifestyle behaviors occurred. These pandemic-related deviations may have influenced participants’ lifestyle patterns and reduced the generalizability of our findings to non-pandemic periods. Fourth, although NHANES employs a complex, multistage probability sampling design with recommended survey weights to ensure nationally representative estimates, our analyses did not incorporate these weights. As a result, the findings should be interpreted with caution and may not be fully generalizable to the U.S. population. Fifth, both the covariates and the lifestyle factors included in the composite score were dichotomized for analysis. Although this approach improved model stability and facilitated interpretation, it inevitably led to a loss of information compared with treating variables in their original forms, and may not fully capture nonlinear or graded associations. Sixth, residual confounding from unmeasured factors cannot be entirely ruled out. Notably, the study does not differentiate between specific etiologies of infertility, each of which could harbor unique risk factors warranting further exploration. In addition, the omission of male infertility factors could lead to potential misclassification as some cases attributed to women may in fact be due to male causes, thus attenuating the observed associations between female lifestyle factors and infertility. Lastly, the lifestyle evaluation criteria were tailored to the U.S. population, necessitating caution when generalizing findings to other populations. In addition, the external dataset focused on women seeking fertility care and only validated the uric acid–infertility association, while further studies with comprehensive lifestyle measures are needed to confirm the lifestyle–infertility association in a broader community.

## 5 Conclusion

Our study highlights the inverse association between multiple healthy lifestyle factors and infertility risk among reproductive-aged women. Serum uric acid was identified as a significant mediator in this relationship, and optimal waist circumference and smoking showed the most pronounced impact. These results suggest the value of comprehensive lifestyle modification as a potential strategy for managing infertility.

## Data Availability

The raw data supporting the conclusions of this article will be made available by the authors, without undue reservation.
