# Supplementary material for: Adherence to a healthy lifestyle in association with female infertility risk: the mediating role of uric acid
Source: Front Nutr. 2025 Oct 10;12:1654495. doi: 10.3389/fnut.2025.1654495 (PMC12549233; doi:10.3389/fnut.2025.1654495)
Supplement: Supplementary file 1 [file Table_1.docx]

**Supplementary Materials**

**Adherence to a healthy lifestyle in association with female infertility risk: the mediating role of uric acid**

**Table of Contents**

[Table S1. Definitions of healthy and unhealthy lifestyle factors for women in this study. 3](#_Toc206861655)

[Table S2. Characteristics of participants with different numbers of healthy lifestyle factors from the National Health and Nutrition Examination Survey. 4](#_Toc206861656)

[Table S3. Associations of each healthy lifestyle factor with risk of infertility. 6](#_Toc206861657)

[Table S4. Associations of different lifestyle scores consisting of five lifestyle scores with risk of infertility. 7](#_Toc206861658)

[Table S5. Association of healthy lifestyle score with risk of infertility after excluding women who reported a history of ovariectomy or hysterectomy. 8](#_Toc206861659)

[Table S6. Association of healthy lifestyle score with risk of infertility after redefining the healthy level of alcohol drinking. 9](#_Toc206861660)

[Table S7. Association of healthy lifestyle score with risk of infertility after propensity score adjustment. 10](#_Toc206861661)

[Table S8. E-values and lower limit of 95% CIs for the association of healthy lifestyle score with risk of infertility. 11](#_Toc206861662)

[Table S9. The coefficients of each healthy lifestyle factor in the logistic regression model. 12](#_Toc206861663)

[Table S10. Association of weighted healthy lifestyle score with risk of infertility. 13](#_Toc206861664)

[Table S11. Association of healthy lifestyle score with serum uric acid. 14](#_Toc206861665)

[Table S12. Association of serum uric acid with risk of infertility. 15](#_Toc206861666)

[Table S13. Baseline characteristics of women in the external validation cohort. 16](#_Toc206861667)

[Table S14. Association of serum uric acid levels with risk of female infertility in the external validation cohort. 17](#_Toc206861668)

[Figure S1. Flow of eligible participants selection. 18](#_Toc206861669)

# Table S1. Definitions of healthy and unhealthy lifestyle factors for women in this study.

| **Factor** | **Healthy level** | **Unhealthy level** |
| --- | --- | --- |
| Tobacco smoking | Current nonsmoking | Current smoking |
| Alcohol drinking | 1-14 g/day | 0 or >14 g/day |
| Physical activity | Moderate-to-vigorous leisure-time physical activity of ≥150 min/week | Moderate-to-vigorous leisure-time physical activity of <150 min/week |
| Diet | Top two-fifths of HEI-2015 score (≥51.77) | Bottom three-fifths of HEI-2015 score (<51.77) |
| Waist circumference | Waist circumference <80 cm | Waist circumference ≥80 cm |
| Sleep duration | 7-9 h | <7 or >9 h |

Abbreviation: HEI, Healthy Eating Index.

# Table S2. Characteristics of participants with different numbers of healthy lifestyle factors from the National Health and Nutrition Examination Survey.

| **Characteristics^a^** | **No. of healthy lifestyle factors** | | | | | ***P*-value^b^** |
| --- | --- | --- | --- | --- | --- | --- |
|  | **0-1** | **2** | **3** | **4** | **5-6** |  |
| No. of participants | 176 | 374 | 600 | 529 | 388 |  |
| Age, years | 33.41 (7.02) | 33.1 (7.08) | 32.9 (7.39) | 32.79 (7.47) | 31.25 (7.28) | <0.001 |
| BMI, kg/m^2^ | 33.27 (8.94) | 33.36 (9.05) | 31.7 (9.05) | 29.25 (7.77) | 24.69 (6.26) | <0.001 |
| Waist circumference, cm | 105.8 (19.54) | 104.85 (20.12) | 100.17 (19.01) | 94.76 (17.01) | 83.45 (14.64) | <0.001 |
| HEI-2015 | 40.95 (6.89) | 42.92 (8.13) | 46.48 (9.77) | 53.92 (11.49) | 59.38 (10.9) | <0.001 |
| Serum uric acid, mg/dL | 4.69 (1.17) | 4.79 (1.11) | 4.61 (1.14) | 4.48 (1.1) | 4.28 (0.91) | <0.001 |
| Race/ethnicity, n (%) |  |  |  |  |  | <0.001 |
| Non-Hispanic white | 76 (43.18) | 129 (34.49) | 189 (31.50) | 184 (34.78) | 169 (43.56) |  |
| Non-Hispanic black | 54 (30.68) | 126 (33.69) | 162 (27.00) | 107 (20.23) | 44 (11.34) |  |
| Mexican American | 14 (7.95) | 46 (12.30) | 99 (16.50) | 96 (18.15) | 44 (11.34) |  |
| Others | 32 (18.18) | 73 (19.52) | 150 (25.00) | 142 (26.84) | 131 (33.76) |  |
| Marital status, n (%) |  |  |  |  |  | <0.001 |
| Married | 55 (31.25) | 157 (41.98) | 279 (46.50) | 255 (48.20) | 197 (50.77) |  |
| Others | 121 (68.75) | 217 (58.02) | 321 (53.50) | 274 (51.80) | 191 (49.23) |  |
| Education attainment, n (%) |  |  |  |  |  | <0.001 |
| Under high school | 48 (27.27) | 63 (16.84) | 81 (13.50) | 47 (8.88) | 13 (3.35) |  |
| High school | 59 (33.52) | 99 (26.47) | 111 (18.50) | 76 (14.37) | 42 (10.82) |  |
| Above high school | 69 (39.20) | 212 (56.68) | 408 (68.00) | 406 (76.75) | 333 (85.82) |  |
| Family PIR, n (%) |  |  |  |  |  | <0.001 |
| <1.3 | 110 (62.50) | 171 (45.72) | 209 (34.83) | 139 (26.28) | 68 (17.53) |  |
| 1.3-<3.5 | 53 (30.11) | 143 (38.24) | 265 (44.17) | 196 (37.05) | 122 (31.44) |  |
| ≥3.5 | 13 (7.39) | 60 (16.04) | 126 (21.00) | 194 (36.67) | 198 (51.03) |  |
| Current nonsmoking, n (%) | 32 (18.18) | 229 (61.23) | 501 (83.50) | 504 (95.27) | 385 (99.23) | <0.001 |
| Low-to-moderate alcohol drinking, n (%) | 79 (44.89) | 266 (71.12) | 526 (87.67) | 491 (92.82) | 378 (97.42) | <0.001 |
| Adequate physical activity, n (%) | 8 (4.55) | 49 (13.10) | 157 (26.17) | 290 (54.82) | 346 (89.18) | <0.001 |
| Healthy diet, n (%) | 6 (3.41) | 38 (10.16) | 152 (25.33) | 308 (58.22) | 323 (83.25) | <0.001 |
| Optimal waist circumference, n (%) | 7 (3.98) | 20 (5.35) | 78 (13.00) | 103 (19.47) | 218 (56.19) | <0.001 |
| Sufficient sleep duration, n (%) | 26 (14.77) | 146 (39.04) | 386 (64.33) | 420 (79.40) | 367 (94.59) | <0.001 |
| Hypertension, n (%) | 54 (30.68) | 91 (24.33) | 122 (20.33) | 69 (13.04) | 30 (7.73) | <0.001 |
| Diabetes, n (%) | 19 (10.80) | 39 (10.43) | 50 (8.33) | 30 (5.67) | 13 (3.35) | <0.001 |
| CVD, n (%) | 10 (5.68) | 9 (2.41) | 20 (3.33) | 4 (0.76) | 2 (0.52) | <0.001 |
| Regular menstrual cycle, n (%) | 144 (81.82) | 328 (87.70) | 541 (90.17) | 486 (91.87) | 363 (93.56) | <0.001 |
| History of pelvic infection, n (%) | 19 (10.80) | 24 (6.42) | 38 (6.33) | 16 (3.02) | 10 (2.58) | <0.001 |
| History of pregnancy, n (%) | 145 (82.39) | 298 (79.68) | 444 (74.00) | 350 (66.16) | 195 (50.26) | <0.001 |
| Taking hormone pills, n (%) | 1 (0.57) | 15 (4.01) | 28 (4.67) | 23 (4.35) | 20 (5.15) | 0.13 |

^a^ Continuous variables were expressed as mean (SD), and categorical variables were presented as number (percentage).

^b^ *P*-values were calculated by one way analysis of variance for continuous variables, and chi-square test for categorical variables.

Abbreviations: BMI, body mass index; CVD, cardiovascular disease; HEI, Healthy Eating Index; PIR, poverty-income ratio.

# Table S3. Associations of each healthy lifestyle factor with risk of infertility.

| **Healthy lifestyle factor** | **OR (95% CI)** |
| --- | --- |
| Current nonsmoking | 0.70 (0.51-0.98) |
| Low-to-moderate drinking | 0.72 (0.53-1.00) |
| Adequate physical activity | 0.79 (0.60-1.04) |
| Optimal waist circumference | 0.61 (0.41-0.89) |
| Healthy diet | 0.91 (0.68-1.19) |
| Sufficient sleep duration | 0.88 (0.67-1.15) |

Data were presented as odds ratio (95% confidence interval). Covariates included in models were shown in the footnote of Table 2, and five lifestyle factors were mutually adjusted for each other.

# Table S4. Associations of different lifestyle scores consisting of five lifestyle scores with risk of infertility.

| **Remained factor** | **Removed factor** | **No. of healthy lifestyle factors** | | | |
| --- | --- | --- | --- | --- | --- |
|  |  | **0-1** | **2** | **3** | **4-5** |
| Alcohol consumption, physical activity, diet, waist circumference, and sleep duration | Smoking | 1.00 (reference) | 0.81 (0.57-1.14) | 0.75 (0.52-1.08) | 0.47 (0.29-0.74) |
| Smoking, physical activity, diet, waist circumference, and sleep duration | Alcohol consumption | 1.00 (reference) | 0.82 (0.58-1.16) | 0.65 (0.44-0.94) | 0.49 (0.31-0.76) |
| Smoking, alcohol consumption, diet, waist circumference, and sleep duration | Physical activity | 1.00 (reference) | 0.67 (0.44-1.02) | 0.56 (0.37-0.85) | 0.46 (0.29-0.72) |
| Smoking, alcohol consumption, physical activity, diet, and sleep duration | Waist circumference | 1.00 (reference) | 0.56 (0.36-0.87) | 0.48 (0.31-0.75) | 0.43 (0.28-0.68) |
| Smoking, alcohol consumption, physical activity, waist circumference, and sleep duration | Diet | 1.00 (reference) | 0.54 (0.35-0.82) | 0.52 (0.35-0.79) | 0.36 (0.23-0.57) |
| Smoking, alcohol consumption, physical activity, diet, and waist circumference | Sleep duration | 1.00 (reference) | 0.54 (0.37-0.78) | 0.58 (0.40-0.84) | 0.37 (0.23-0.57) |

Data were presented as odds ratio (95% confidence interval). Covariates included in models were shown in the footnote of Table 2.

# Table S5. Association of healthy lifestyle score with risk of infertility after excluding women who reported a history of ovariectomy or hysterectomy.

| **Variable** | **No. of healthy lifestyle factors** | | | | | **Each additional healthy lifestyle factor** |
| --- | --- | --- | --- | --- | --- | --- |
|  | **0-1** | **2** | **3** | **4** | **5-6** |  |
| Case/total (%) | 37/164 (22.56) | 56/351 (15.95) | 78/569 (13.71) | 71/502 (14.14) | 33/375 (8.8) |  |
| Crude model | 1.00 (reference) | 0.65 (0.41-1.04) | 0.55 (0.35-0.85) | 0.57 (0.36-0.89) | 0.33 (0.20-0.55) | 0.81 (0.73-0.89) |
| Model 1^a^ | 1.00 (reference) | 0.67 (0.42-1.08) | 0.56 (0.36-0.88) | 0.59 (0.38-0.93) | 0.36 (0.22-0.61) | 0.82 (0.74-0.91) |
| Model 2^b^ | 1.00 (reference) | 0.62 (0.38-1.00) | 0.51 (0.32-0.81) | 0.52 (0.32-0.86) | 0.31 (0.18-0.55) | 0.80 (0.71-0.89) |

^a^ Model 1 was adjusted for age (<30, ≥30 years) and race/ethnicity (non-Hispanic white, others).

^b^ Model 2 was further adjusted for marital status (married, others), family poverty-income ratio (<3.5, ≥3.5), education attainment (above high school, high school and below), hypertension (yes, no), diabetes (yes, no), CVD (yes, no), regular menstrual cycle (yes, no), history of pelvic infection (yes, no), history of pregnancy (yes, no), and taking hormone pills (yes, no).

# Table S6. Association of healthy lifestyle score with risk of infertility after redefining the healthy level of alcohol drinking.

| **Variable** | **No. of healthy lifestyle factors** | | | | | **Each additional healthy lifestyle factor** |
| --- | --- | --- | --- | --- | --- | --- |
|  | **0-1** | **2** | **3** | **4** | **5-6** |  |
| Case/total (%) | 33/138 (23.91) | 55/345 (15.94) | 89/628 (14.17) | 77/547 (14.08) | 38/409 (9.29) |  |
| Crude model | 1.00 (reference) | 0.60 (0.37-0.99) | 0.53 (0.34-0.83) | 0.52 (0.33-0.83) | 0.33 (0.19-0.55) | 0.81 (0.73-0.89) |
| Model 1^a^ | 1.00 (reference) | 0.60 (0.37-0.99) | 0.53 (0.34-0.85) | 0.53 (0.33-0.84) | 0.35 (0.21-0.58) | 0.82 (0.74-0.90) |
| Model 2^b^ | 1.00 (reference) | 0.54 (0.33-0.89) | 0.46 (0.28-0.74) | 0.44 (0.27-0.72) | 0.28 (0.16-0.49) | 0.78 (0.69-0.87) |

^a^ Model 1 was adjusted for age (<30, ≥30 years) and race/ethnicity (non-Hispanic white, others).

^b^ Model 2 was further adjusted for marital status (married, others), family poverty-income ratio (<3.5, ≥3.5), education attainment (above high school, high school and below), hypertension (yes, no), diabetes (yes, no), CVD (yes, no), regular menstrual cycle (yes, no), history of pelvic infection (yes, no), history of pregnancy (yes, no), and taking hormone pills (yes, no).

# Table S7. Association of healthy lifestyle score with risk of infertility after propensity score adjustment.

| **Variable** | **No. of healthy lifestyle factors** | | | | | **Each additional healthy lifestyle factor** |
| --- | --- | --- | --- | --- | --- | --- |
|  | **0-1** | **2** | **3** | **4** | **5-6** |  |
| Case/total (%) | 40/176 (22.73) | 61/374 (16.31) | 83/600 (13.83) | 73/529 (13.8) | 35/388 (9.02) |  |
| Crude model | 1.00 (reference) | 0.66 (0.43-1.04) | 0.55 (0.36-0.84) | 0.54 (0.36-0.84) | 0.34 (0.20-0.55) | 0.80 (0.73-0.89) |
| PS-adjusted model^a^ | 1.00 (reference) | 0.64 (0.41-1.02) | 0.53 (0.35-0.82) | 0.55 (0.36-0.86) | 0.35 (0.21-0.58) | 0.82 (0.74-0.90) |

^a^ PS was defined as the probability of being infertility given a set of covariates, including age (<30, ≥30 years), race/ethnicity (non-Hispanic white, others), marital status (married, others), family poverty-income ratio (<3.5, ≥3.5), education attainment (above high school, high school and below), hypertension (yes, no), diabetes (yes, no), CVD (yes, no), regular menstrual cycle (yes, no), history of pelvic infection (yes, no), history of pregnancy (yes, no), and taking hormone pills (yes, no).

Abbreviation: PS, propensity score.

# Table S8. E-values and lower limit of 95% CIs for the association of healthy lifestyle score with risk of infertility.

| **Variable** | **OR (95% CI)** | ***E*-value** |
| --- | --- | --- |
| 0-1 healthy lifestyle factors | 1.00 (ref.) | – |
| 2 healthy lifestyle factors | 0.60 (0.38-0.96) | 2.72 |
| 3 healthy lifestyle factors | 0.49 (0.31-0.76) | 3.50 |
| 4 healthy lifestyle factors | 0.49 (0.30-0.78) | 3.50 |
| 5-6 healthy lifestyle factors | 0.30 (0.17-0.52) | 6.12 |
| Each additional healthy lifestyle factor | 0.78 (0.70-0.88) | 1.88 |

# Table S9. The coefficients of each healthy lifestyle factor in the logistic regression model.

| **Healthy lifestyle factor in the model** | **β coefficient** | **Weighted β coefficient** |
| --- | --- | --- |
| Tobacco smoking (healthy vs unhealthy) | –0.36 | 0.22 |
| Alcohol drinking (healthy vs unhealthy) | –0.33 | 0.20 |
| Physical activity (healthy vs unhealthy) | –0.24 | 0.14 |
| Diet (healthy vs unhealthy) | –0.10 | 0.06 |
| Waist circumference (healthy vs unhealthy) | –0.49 | 0.30 |
| Sleep duration (healthy vs unhealthy) | –0.13 | 0.08 |
| Total | –1.64 | 1.00 |

# Table S10. Association of weighted healthy lifestyle score with risk of infertility.

| **Variable** | **Quartiles of weighted healthy lifestyle score** | | | | **Each additional score** |
| --- | --- | --- | --- | --- | --- |
|  | **Quartile 1 (lowest)** | **Quartile 2** | **Quartile 3** | **Quartile 4 (highest)** |  |
| Case/total (%) | 110/600 (18.33) | 59/439 (13.44) | 78/513 (15.2) | 45/515 (8.74) |  |
| Crude model | 1.00 (reference) | 0.69 (0.49-0.97) | 0.80 (0.58-1.10) | 0.43 (0.29-0.61) | 0.77 (0.69-0.85) |
| Model 1^a^ | 1.00 (reference) | 0.70 (0.49-0.99) | 0.82 (0.60-1.13) | 0.47 (0.32-0.68) | 0.79 (0.71-0.87) |
| Model 2^b^ | 1.00 (reference) | 0.66 (0.46-0.94) | 0.78 (0.56-1.10) | 0.44 (0.29-0.66) | 0.76 (0.68-0.85) |

^a^ Model 1 was adjusted for age (<30, ≥30 years) and race/ethnicity (non-Hispanic white, others).

^b^ Model 2 was further adjusted for marital status (married, others), family poverty-income ratio (<3.5, ≥3.5), education attainment (above high school, high school and below), hypertension (yes, no), diabetes (yes, no), CVD (yes, no), regular menstrual cycle (yes, no), history of pelvic infection (yes, no), history of pregnancy (yes, no), and taking hormone pills (yes, no).

# Table S11. Association of healthy lifestyle score with serum uric acid.

| **healthy lifestyle score** | **Crude model** | **Model 1^a^** | **Model 2^b^** |
| --- | --- | --- | --- |
| No. of healthy lifestyle factor |  |  |  |
| 0-1 | 0.00 (reference) | 0.00 (reference) | 0.00 (reference) |
| 2 | 0.10 (–0.10, 0.29) | 0.10 (–0.10, 0.29) | 0.11 (–0.08, 0.31) |
| 3 | –0.09 (–0.27, 0.09) | –0.08 (–0.27, 0.10) | –0.05 (–0.23, 0.13) |
| 4 | –0.21 (–0.40, –0.03) | –0.21 (–0.40, –0.03) | –0.15 (–0.34, 0.04) |
| 5-6 | –0.42 (–0.61, –0.22) | –0.43 (–0.62, –0.24) | –0.35 (–0.55, –0.15) |
| Each additional healthy lifestyle factor | –0.12 (–0.16, –0.09) | –0.13 (–0.16, –0.09) | –0.11 (–0.15, –0.07) |

^a^ Model 1 was adjusted for age (<30, ≥30 years) and race/ethnicity (non-Hispanic white, others).

^b^ Model 2 was further adjusted for marital status (married, others), family poverty-income ratio (<3.5, ≥3.5), education attainment (above high school, high school and below), hypertension (yes, no), diabetes (yes, no), and CVD (yes, no).

# Table S12. Association of serum uric acid with risk of infertility.

| **Variable** | **Quartiles of serum uric acid** | | |  | ***P*-trend** | **Per SD increment** |
| --- | --- | --- | --- | --- | --- | --- |
|  | **Quartile 1** | **Quartile 2** | **Quartile 3** | **Quartile 4** |  |  |
| Range | <3.9 | 3.9-<4.6 | 4.6-<5.3 | ≥5.3 |  |  |
| Median | 3.4 | 4.2 | 4.9 | 5.9 |  |  |
| Case/control (%) | 64/561 (11.41) | 62/534 (11.61) | 71/495 (14.34) | 95/477 (19.92) |  |  |
| Crude model | 1.00 (reference) | 1.02 (0.70-1.48) | 1.30 (0.91-1.87) | 1.93 (1.37-2.73) | <0.001 | 1.30 (1.15-1.46) |
| Model 1^a^ | 1.00 (reference) | 1.07 (0.74-1.56) | 1.38 (0.96-1.98) | 1.96 (1.39-2.78) | <0.001 | 1.30 (1.16-1.47) |
| Model 2^b^ | 1.00 (reference) | 1.04 (0.71-1.52) | 1.34 (0.93-1.95) | 1.98 (1.39-2.85) | <0.001 | 1.31 (1.16-1.49) |

^a^ Model 1 was adjusted for age (<30, ≥30 years) and race/ethnicity (non-Hispanic white, others).

^b^ Model 2 was further adjusted for marital status (married, others), family poverty-income ratio (<3.5, ≥3.5), education attainment (above high school, high school and below), hypertension (yes, no), diabetes (yes, no), CVD (yes, no), regular menstrual cycle (yes, no), history of pelvic infection (yes, no), history of pregnancy (yes, no), and taking hormone pills (yes, no).

Abbreviations: SD, standard deviation.

# Table S13. Baseline characteristics of women in the external validation cohort.

| **Characteristics**^a^ | **Non-infertility (N=1854)** | **Infertility (N=32047)** | ***P*-value** |
| --- | --- | --- | --- |
| Age (years) | 30 (27-35) | 30 (27-34) | 0.198 |
| BMI (kg/m^2^) | 21.3 (19.7-23.3) | 21.6 (19.9-23.9) | <0.001 |
| Infertility duration (years) | 0.5 (0.4-0.7) | 3.0 (2.0-5.0) | <0.001 |
| Nulligravida, n (%) | 258 (13.9) | 13033 (40.7) | <0.001 |
| Nulliparity, n (%) | 1191 (64.2) | 21613 (67.5) | 0.004 |
| Current smoking, n (%) | 25 (1.4) | 486 (1.5) | 0.562 |
| SBP (mmHG) | 110 (102-119) | 111 (102-120) | 0.001 |
| DBP (mmHG) | 66 (60-73) | 67 (61-74) | 0.023 |
| Year of treatment, n (%) |  |  | <0.001 |
| 2014-2016 | 431 (23.3) | 8712 (27.2) |  |
| 2017-2019 | 666 (35.9) | 12754 (39.8) |  |
| 2020-2022 | 757 (40.8) | 10561 (33) |  |
| Serum uric acid (µmol/L) | 282 (247-322) | 285 (248-329) | 0.007 |

^a^ Continuous variables were expressed as median (interquartile range), and categorical variables were presented as number (percentage).

Abbreviations: BMI, body mass index; SBP, systolic blood pressure; DBP, diastolic blood pressure.

# Table S14. Association of serum uric acid levels with risk of female infertility in the external validation cohort.

|  | **Crude OR**  **(95% CI)** | ***P*-value** | **Adjusted OR**  **(95% CI)**^a^ | ***P*-value** |
| --- | --- | --- | --- | --- |
| Uric acid (continuous) | 1.001 (1.000-1.002) | 0.002 | 1.001 (1.000-1.002) | 0.007 |
| Uric acid (quartiles) |  |  |  |  |
| Q1 (≤ 248 µmol/L) | 1 (Ref) |  | 1 (Ref) |  |
| Q2 (248-285 µmol/L) | 0.97 (0.85-1.11) | 0.652 | 0.97 (0.85-1.11) | 0.669 |
| Q3 (285-328 µmol/L) | 0.96 (0.85-1.10) | 0.565 | 0.96 (0.84-1.10) | 0.546 |
| Q4 (>328 µmol/L) | 1.21 (1.06-1.39) | 0.006 | 1.19 (1.04-1.37) | 0.013 |

^a^ Adjusted for age, gravidity, parity, smoking status, systolic and diastolic blood pressure, and year of treatment.

Abbreviations: OR, odds ratio; CI,


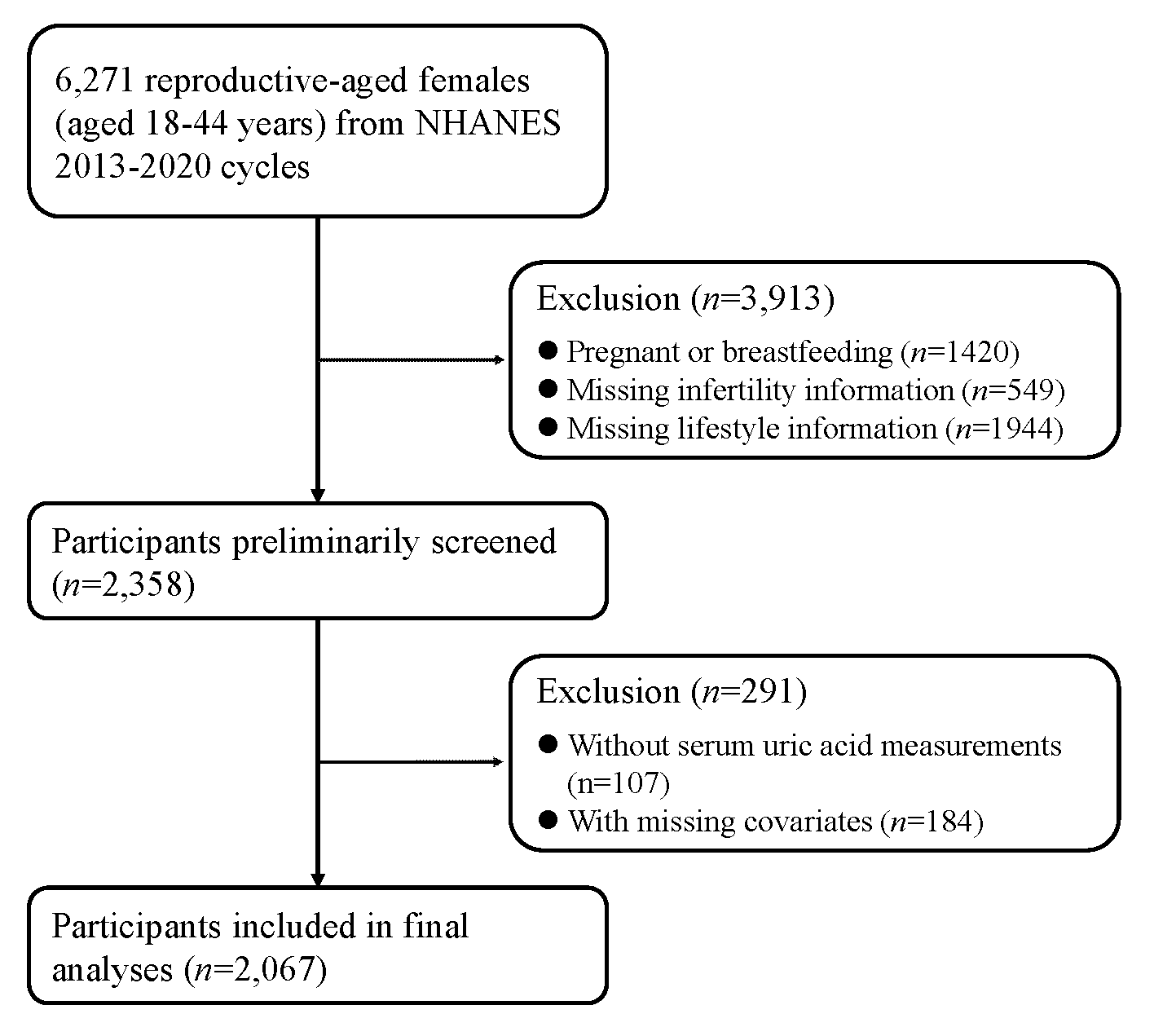


# Figure S1. Flow of eligible participants selection.

Abbreviations: NHANES, National Health and Nutrition Examination Survey.
